# Real-world use of an artificial intelligence–powered clinical decision support tool for ovarian stimulation

**DOI:** 10.1016/j.xfre.2025.01.015

**Published:** 2025-01-28

**Authors:** Cameron J. Bixby, Bradley Miller

**Affiliations:** a Henry Ford Providence Hospital, Michigan State University College of Human Medicine, Southfield, Michigan; b Reproductive Medicine Associates of Michigan, Reproductive Endocrinology, Troy, Michigan

**Keywords:** Artificial intelligence, machine learning, ovarian stimulation, ART

## Abstract

**Objective:**

To understand how treatment decisions and patient outcomes change with physician utilization of artificial intelligence (AI) to help determine follicle-stimulating hormone (FSH) starting dose and trigger injection timing during ovarian stimulation.

**Design:**

Retrospective cohort study with historical controls.

**Subjects:**

Patients undergoing ovarian stimulation by multiple physicians at one in vitro fertilization clinic in the United States.

**Exposure:**

A total of 292 patients were treated between December 2022 and December 2023 with adjunctive clinician use of AI to help select starting dose of FSH and timing of the trigger injection. These were matched to 292 historical control patients treated between May 2019 and May 2022 by the same physicians without AI.

**Main Outcome Measures:**

The primary endpoints were the starting FSH dose, total FSH dose, and number of metaphase II (MII) oocytes retrieved at the end of stimulation.

**Results:**

The use of AI did not introduce any adverse events. After patient matching, there were no statistically significant differences in age, body mass index, antimüllerian hormone, or antral follicle count between the treatment and control groups. Comparing the treatment arm with the control arm, the average number of MII oocytes was 11.17 vs. 11.25, the average starting FSH dose was 397.09 IU vs. 443.84 IU, and the average total FSH dose was 4,181.77 IU vs. 4,654.71 IU.

**Conclusion:**

Physician use of AI helped significantly reduce the starting and total FSH doses prescribed to patients without adversely affecting MII outcomes, indicating the potential use of AI in lowering in vitro fertilization costs to patients.

In vitro fertilization (IVF) is a common method of assisted reproductive technology and, in 2021, accounted for 2.3% of all infants born in the United States (1). The continued increase in IVF treatment brings to the forefront efforts to improve efficiency and outcomes for couples seeking to conceive. However, many decisions are subjective in the IVF process, especially regarding the starting and total follicle-stimulating hormone (FSH) doses prescribed to patients for ovarian stimulation. Insufficient FSH dosing affects the overall growth of ovarian follicles and, therefore, the total number of oocytes at time of retrieval (2). On the contrary, excessive FSH can lead to complications such as ovarian hyperstimulation syndrome (OHSS). Not only is FSH dosing subjective, but also the optimal trigger injection timing for mature oocyte (metaphase II [MII]) retrieval, which is one of the most important decisions in IVF. The subjective nature of these critical decisions is due to varying baseline patient criteria, differing techniques among clinical practices, and physician expertise. Therefore, the growing use of artificial intelligence (AI) has begun to shape the landscape of IVF to better assist physicians with these important decisions.

With the enormous amount of electronic medical record data available to modern-day clinicians, core AI algorithms can be used to help shape clinical decision-making (3). For example, Hariton et al. (4) used a machine learning algorithm to determine optimal timing of trigger injections to increase the average number of fertilized oocytes (2 pronuclei [2PNs]) and the total number of blastocysts compared with a physician’s decision alone. Additionally, Fanton et al. (5), in a retrospective analysis, incorporated interpretable machine learning models using linear regression to help make efficient day-to-day trigger injection decisions, which revealed that fewer MII oocytes, 2PNs, and usable blastocysts were found with early or late triggers. This further highlights the importance of trigger injection timing, and with the expansion of AI and predictive models, this can better support clinical decision-making. Although prior studies have retrospectively estimated the benefit of using AI for treatment decisions during ovarian stimulation, there is a need for more studies that prospectively use and evaluate these AI models.

In this study, an AI clinical decision support software was used to assist with the appropriate selection of the starting dose of FSH as well as the decision of when to administer the trigger injection by making predictions of MII oocytes for different treatment options. This AI software was used commercially for 1 year by multiple physicians at a large private practice in the United States. This study is an analysis of patients treated with this AI tool to understand how treatment and patient outcomes change with the real-world clinical use of AI for ovarian stimulation. It consisted of patients seen by physicians using this AI software compared with matched historical control patients seen by the same physicians where AI was not used. The primary goal of this study was to evaluate whether treatment decisions and patient MII outcomes changed with adjunctive use of AI software to help determine the starting and total doses of FSH during ovarian stimulation.

## Materials and methods

### Ethics approval

The Western-Copernicus Group Institutional Review Board provided a full exemption for this study.

### AI software

The AI software used in this study was Stim Assist (Alife Health, Inc., San Francisco, CA). Stim Assist is a clinical decision support software to optimize treatment decisions during ovarian stimulation by providing clinicians with adjunctive predictions about how treatment decisions affect MII outcomes. The software includes two previously published AI algorithms: a Starting Dose Tool (6) and a Trigger Tool (5). Although the primary focus of this study is on the real-world clinical outcomes and dosing trends observed after the deployment of the AI software, rather than the detailed mechanisms of the Starting Dose Tool and Trigger Tool algorithm development, we have provided a high-level description of each model in the following.

The Starting Dose Tool is intended to be used before the start of stimulation to help guide clinicians on their decision of the starting dose of FSH. The model uses a patient’s baseline antimüllerian hormone (AMH) level, baseline antral follicle count (AFC), and body mass index to create a dose-response curve relating the starting dose of FSH to a predicted number of MII oocytes retrieved. The model was trained on a total of 18,591 IVF cycles from three clinics in the United States, between 2014 and 2020. The model uses a K-nearest neighbors (KNN) algorithm to identify the 100 most similar patients to a patient of interest, which are then used to fit a curve relating MII oocytes to the starting dose of FSH. In this model, the total starting FSH dose was calculated as the sum of pure FSH (e.g., Gonal-F or Follistim) plus the FSH component of FSH/luteinizing hormone medication (e.g., Menopur) (6).

The Trigger Tool is intended to be used throughout the stimulation cycle to predict the number of MII oocytes to be retrieved if triggered today, tomorrow, or in 2 days. The Trigger Tool also makes predictions of estradiol (E2) tomorrow, 2 days, and 3 days in the future. The model was trained on 30,278 IVF cycles from three IVF clinics in the United States, between 2014 and 2020. Predictions are made through a set of interpretable linear regression models, which take in a patient’s latest E2 and follicle measurements (5).

Stim Assist is intended to be used on patients undergoing conventional IVF cycles for both autologous and donor patients. Stim Assist was fully integrated with the electronic medical record, without the need for manual data entry. When opening the software, a list of patients being actively treated on the current day was automatically populated, with the additional ability to search for specific patients via their patient identification. The AI algorithms generate their predictions in real time (<1 second) to ensure no delays in clinical workflow.

### Study design and participants

This study was an observational, postmarket study conducted by multiple physicians at Reproductive Medicine Associates of Michigan, in Troy, Michigan. The physicians using this tool during the study period were highly experienced, collectively amassing >50 years of clinical practice. There were a total of 292 patients treated with adjunctive use of the AI software included in the treatment arm between December 2022 and December 2023. The control arm consisted of retrospective historical control patients who had treatment without the AI software between May 2019 and May 2022. All patients undergoing conventional autologous IVF cycles during the study period were included.

During the study period, clinicians used the AI software before the start of stimulation to help guide starting FSH dose selection and during stimulation to help guide the decision of whether to give the trigger injection or to push additional days. All treatment decisions were ultimately made by the physician because the AI software only provided adjunctive predictions rather than specific dosing or trigger injection timing recommendations.

### Statistical analysis

To compare the treatment arm patients with the historical controls, we performed 1-to-1 patient matching on the basis of age, baseline AFC, and baseline AMH. For patients in the treatment arm with missing AMH and AFC, these values were imputed using a KNN imputer, trained on data from 23,000 prior IVF patients. All variables were standardized by subtracting the mean and dividing by the standard deviation across all patients, ensuring comparability. To create the matched historical control group, each patient in the treatment arm was matched, with replacement, to their nearest historical control patient using a KNN algorithm, resulting in 292 matched historical controls.

The primary endpoints of the number of MII oocytes, starting FSH dose, and total FSH dose were compared between the two groups. The peak E2 level on the day of trigger injection was also compared between groups. A *t*-test was conducted to assess the difference in means between the two groups, with a significance threshold set at *P*<.05. Additionally, a subanalysis was performed to compare these outcomes for patients aged <35, 35–40, and ≥40 years.

## Results

There were a total of 292 patients where the AI software was used for treatment, whereas the control group consisted of 292 matched, historical control patients treated without use of the AI software. There were 49 patients (17%) in the treatment arm of the study who were missing AMH or AFC and had these values imputed. After patient matching, there were no significant differences in age, AMH, AFC, or body mass index between the treatment and control arms. There were no reported cases of severe OHSS in either the treatment or historical control group.

The overall average starting FSH dose, total FSH dose, and average number of MII oocytes in the control vs. treatment arm are summarized in Table 1. The overall average number of MII oocytes collected in the control vs. treatment arm was 11.25 vs. 11.17 (*P*=.90). The overall average starting FSH dose in the control vs. treatment arm was 443.84 IU vs. 397.09 IU (*P*<.01), whereas the overall average total FSH dose in the control vs. treatment arm was 4,654.71 IU vs. 4,181.77 IU (*P*<.01).

**Table 1:** Comparison of the average number of metaphase II oocytes, starting follicle-stimulating hormone dose, and total follicle-stimulating hormone dose outcomes in the control arm compared with the artificial intelligence software treatment arm.

Group	Average number of MII oocytes	Average starting FSH dose (IU)	Average total FSH dose (IU)
Control	11.25	443.84	4,654.71
Treatment	11.18	397.09	4,181.77
*P* value	.90	<.01	<.01

*Note:* FSH = follicle-stimulating hormone; MII = metaphase II.

The average number of MII oocytes, starting FSH dose, and total FSH dose observed by age groups are summarized in Table 2. The average number of MII oocytes in the control vs. treatment arm in patients aged <35 years was 15.27 vs. 13.75 (*P*=.15). The average starting and total FSH doses in the control vs. treatment arm in patients aged <35 years were 387.50 IU vs. 351.14 IU (*P*<.01) and 4,012.12 IU vs. 3,566.11 IU (*P*<.01), respectively. The average number of MII oocytes in the control vs. treatment arm in patients aged 35–40 years was 8.28 vs. 9.33 (*P*=.19). The average starting and total FSH doses in the control vs. treatment arm in patients aged 35–40 years were 488.95 IU vs. 425.00 IU (*P*<.01) and 5,159.30 IU vs. 4,637.79 IU (*P*<.01), respectively. Lastly, the average number of MII oocytes in the control vs. treatment arm in patients aged ≥40 years was 6.55 vs. 7.84 (*P*=.32). The average starting and total FSH doses in the control vs. treatment arm in patients aged ≥40 years were 495.97 IU vs. 476.51 IU (*P*=.32) and 5,291.13 IU vs. 4,905.65 IU (*P*=.34), respectively.

**Table 2:** Average number of metaphase II oocytes, starting follicle-stimulating hormone dose, and total follicle-stimulating hormone dose stratified by age compared between the control and artificial intelligence software treatment arms.

Group	<35 y (N =132)			35–40 y (N = 129)			≥40 y (N = 31)		
	Average number of MII oocytes	Average starting FSH dose (IU)	Average total FSH dose (IU)	Average number of MII oocytes	Average starting FSH dose (IU)	Average total FSH dose (IU)	Average number of MII oocytes	Average starting FSH dose (IU)	Average total FSH dose (IU)
Control	15.27	387.50	4,012.12	8.28	488.95	5,159.30	6.55	495.97	5,291.13
Treatment	13.75	351.14	3,566.11	9.33	425.00	4,637.79	7.84	476.61	4,905.65
*P* value	.15	<.01	<.01	.19	<.01	<.01	.21	.32	.21

*Note:* FSH = follicle-stimulating hormone; MII = metaphase II.

The average E2 levels on the day on trigger injection in the control and treatment arms were 2,564 and 2,626 pg/mL (*P*=.62), respectively. The average E2 levels on the day of trigger in the control and treatment arms in patients aged <35 years were 2,959 and 3,019 pg/mL (*P*=.75), respectively; those in patients aged 35–40 years were 2,186 and 2,348 pg/mL (*P*=.34), respectively; and those in patients aged ≥40 years were 2,458 and 2,116 pg/mL (*P*=.51), respectively.

Lastly, we ran a sensitivity analysis by removing all patients who were missing AMH or AFC from the study, which resulted in 243 patients in the treatment group and 243 matched controls (Table 3, Table 4 and 4). In this analysis, the overall average number of MII oocytes collected in the control vs. treatment arm was 11.34 vs. 11.12 (*P*=.76). The overall average starting FSH dose in the control vs. treatment arm was 445.99 IU vs. 396.30 IU (*P*<.01), whereas the overall average total FSH dose in the control vs. treatment arm was 4,591.87 IU vs. 4,199.70 IU (*P*<.01).

**Table 3:** Sensitivity analysis to remove patients with missing baselines: comparison of the average number of metaphase II oocytes, starting follicle-stimulating hormone dose, and total follicle-stimulating hormone dose outcomes in the control arm compared with the artificial intelligence software treatment arm.

Group	Average number of MII oocytes	Average starting FSH dose (IU)	Average total FSH dose (IU)
Control	11.12	445.99	4,591.87
Treatment	11.34	396.30	4,199.70
*P* value	.76	<.01	<.01

*Note:* FSH = follicle-stimulating hormone; MII = metaphase II.

**Table 4:** Sensitivity analysis to remove patients with missing baselines: average number of metaphase II oocytes, starting follicle-stimulating hormone dose, and total follicle-stimulating hormone dose stratified by age compared between the control and artificial intelligence software treatment arms.

Group	<35 y (N =132)			35–40 y (N = 129)			≥40 y (N = 31)		
	Average number of MII oocytes	Average starting FSH dose (IU)	Average total FSH dose (IU)	Average number of MII oocytes	Average starting FSH dose (IU)	Average total FSH dose (IU)	Average number of MII oocytes	Average starting FSH dose (IU)	Average total FSH dose (IU)
Control	15.78	382.35	3,917.40	8.09	491.22	5,027.70	6.47	495.0	5,272.5
Treatment	14.34	347.06	3,528.21	9.48	420.27	4,622.97	8.03	475.0	4,916.67
*P* value	.25	<.01	.02	.10	<.01	<.01	.24	.32	.27

*Note:* FSH = follicle-stimulating hormone; MII = metaphase II.

## Discussion

The use of the AI software in this clinical study demonstrated its efficacy in reducing overall starting FSH and total FSH dosing during ovarian stimulation without reducing laboratory outcomes, which was the primary endpoint. Overall, when comparing the treatment arm with the control arm, there was a significant decrease in average starting and total FSH doses, resulting in no significant change in mature egg outcomes. There were no cases of moderate or severe OHSS noted in the study participant’s treatment course. The clinicians involved in the study were highly experienced, indicating that the AI software can effectively support even seasoned clinicians in decision-making. Overall, these results suggest that AI could help clinicians optimize their patients’ outcomes while reducing medication used, saving money, and helping reduce pharmaceutical waste. This is extremely important in a medical field where access to fertility care is challenging for so many patients, often due to cost.

When separating patients by age groups, there was a significant decrease in average starting and total FSH doses except for the group of patients aged ≥40 years noted in Table 2. Additionally, there were similar average numbers of MII oocytes noted for both the treatment and control groups (Table 1). Although not statistically significant, there was a slight increase in the total average number of MII oocytes in the groups of patients aged 35–40 and ≥40 years (Table 2, Table 4 and 4), along with a slight decrease in patients aged <35 years. These findings suggest that the AI software tool has the potential to individualize FSH dosing, improving the number of MII oocytes collected in age groups with typically lower ovarian response, while promoting safer outcomes in higher responders. However, given the lack of statistical significance, these observations should be further investigated in future studies with larger sample sizes.

It is widely known that baseline AMH level and AFC, along with the patient’s age, can be used as a predictive measure of both hyposresponses and excessive responses to ovarian stimulation (7). Additionally, Sadruddin et al. (8) had concluded that AMH is a superior indicator of the ovarian stimulation response, which again was used in our baseline characteristics when selecting patients for comparison in this study. Many clinicians will make treatment decisions with regard to starting FSH dose on the basis of these factors, although it varies by expertise and training. In this study, we used all three of these factors when matching the historical control patients to the treated subjects, and the overall average starting and total FSH doses were significantly lower (Table 1). Therefore, this highlights the further importance of not only individualized FSH dosing but the ability of the algorithm to assist in that process of individualized treatment.

The importance of the starting and overall FSH doses cannot be understated especially when considering ideal ovarian response with stimulation. For those who are considered hyporesponders to ovarian stimulation, higher doses of FSH such as 300–600 IU per day have not shown any real benefit (9). The highest average starting FSH dose in our study was 473.78 IU for patients aged ≥40 years in the treatment arm (Table 2), whereas the overall starting FSH dose for all groups in the treatment arm was 397.09 IU (Table 1), which was lower than that in the control group. Therefore, the results in this study can help pave the way for further use of AI-driven algorithms to help personalize ideal FSH treatment for patients and help identify which patients may benefit from lower doses of medication.

The average number of MII oocytes collected in our study was comparable between the treatment and control groups (Table 1). However, the average numbers of collected MII oocytes were similar at lower FSH doses than in the control. This is further supported by another algorithm model–based study by Correa et al. (10), which showed that most patients with 10–15 MII oocytes collected had FSH dosing between 251 and 300 IU. Although this study did not investigate the postfertilization outcomes such as 2PNs, blastocysts, or live birth rates, as the average number of MII oocytes collected increases, the number of blastocysts and chance of reaching a live birth also increase on average (11). Therefore, the use of MII oocytes as an endpoint is a suitable and meaningful proxy for the effectiveness of ovarian stimulation strategies. Furthermore, postfertilization outcomes will depend on external factors such as sperm quality and laboratory protocol, which are not considered in the AI software, which was designed specifically to optimize the number of MII oocytes. Future studies on this AI software should evaluate the impact on downstream outcomes such as blastocyst formation, euploid rates, and live birth rates to further validate the clinical utility.

Individualized FSH dosing has the ability to not only improve controlled ovarian stimulation outcomes with the number of oocytes collected but also reduce the risks of OHSS. There were no exclusions in this study; thus, there were no separation of those who would be considered poor responders, normal responders, or hyperresponders with assisted reproductive technology. Some data suggest that for those considered hyperresponders (AFC, >15), a 150-IU daily dose of FSH significantly increase the number of oocytes collected and reduce the risk of OHSS, whereas those who are poor or normal responders are recommended to have a starting FSH dose of 150–225 or 225–300 IU (12). The highest starting and total FSH dosing in this study was noted in the group of patients aged ≥40 years, which is to be expected, however, the algorithm still lowered the amount of FSH dosing than that in the control group (Table 2). We also observed no adverse outcomes (moderate or severe OHSS) when using the AI software regardless of the predicted types of responders included throughout the treatment group. Therefore, with the use of the algorithm, patients were not inherently at increased risk of OHSS. Other adverse outcomes, such as lack of blastocyst development or inadequate response to treatment, were not tracked in this study because the primary aim of this analysis was to evaluate the AI software’s impact on the number of MII oocytes retrieved and the reduction in gonadotropin usage, rather than broader outcome measures such as blastulation rate or response categorization. These additional adverse outcomes should be the focus of future postmarket analyses.

We analyzed the E2 levels on the day of trigger injection to explore whether differences in dosing were associated with lower E2 levels. No statistically significant differences in the E2 levels were observed between the treatment and control groups. Among patients aged ≥40 years, there was a trend toward lower E2 levels in the treatment group with a higher number of MII oocytes retrieved, although this was not significant. These findings suggest that the AI tool optimizes FSH dosing without substantially altering the E2 levels; however, further studies are needed to determine whether such trends could become significant in larger cohorts. This is particularly relevant to understanding the potential role of AI-guided dosing in reducing OHSS risk because lower E2 levels have been associated with a reduced likelihood of OHSS (13).

We ran a sensitivity analysis to understand the effects of imputation of missing AMH and AFC in our patient matching analysis by removing all patients from the treatment and control groups with missing baselines. We found that the results were very similar after removing these patients, suggesting that the inclusion of imputed data did not materially impact the overall outcomes and, thus, was a valid approach for patient matching.

The strengths of this clinical study include that the AI software was used in a clinical setting and prospectively in nature, whereas most previous studies evaluating potential AI benefit do so in a retrospective manner without actual tool use. This system also demonstrated its efficacy in reducing overall starting FSH and total FSH dosing during ovarian stimulation while not introducing adverse effects during treatment or reducing clinical outcomes. Additionally, patients in the control group were matched on a 1-to-1 basis to the treatment group using criteria such as, age, baseline AMH, and baseline antral follicles. The limitations of this study include that it was conducted at a single IVF clinic and it was not a randomized, controlled trial. Therefore, future prospective studies using larger populations and a diverse set of clinics should be conducted using this AI software to generate results that can be generalizable to a larger population. Additionally, although the underlying algorithms of the AI software were trained on extensive data sets to incorporate a broad range of patient demographics and clinical practices, it is possible that the training data did not encompass all possible clinical scenarios. It is possible that AI models trained on different data sets could have led to different findings. Lastly, physicians with varying training backgrounds, experience levels, and treatment strategies may interpret the AI-generated predictions differently and use the tool in diverse ways, which should be evaluated in future follow-on studies.

## Conclusion

Our study highlights the abilities of an AI software to help guide clinicians in their decisions regarding FSH dosing and trigger timing throughout IVF treatment. This system was able to help lower starting and total FSH dosing overall than that in patients treated without use of AI guidance. Through this system, clinicians can further individualize treatment for patients and safely optimize ovarian stimulation rather than relying on varied expertise and training alone.

## CRediT Authorship Contribution Statement

**Cameron J. Bixby:** Writing – review & editing, Writing – original draft, Formal analysis. **Bradley Miller:** Writing – review & editing, Validation, Resources, Project administration, Data curation, Conceptualization.

## Declaration of Interests

C.J.B. has nothing to disclose. B.M. has nothing to disclose.
